# Supplementary material for: Alteration of the Nucleotide Excision Repair (NER) Pathway in Soft Tissue Sarcoma
Source: Int J Mol Sci. 2022 Jul 28;23(15):8360. doi: 10.3390/ijms23158360 (PMC9369086; doi:10.3390/ijms23158360)
Supplement: Supplementary file 1 [file ijms-23-08360-s001.zip › ijms-1751630-supplementary.pdf]

Supplementary Materials

In order to validate the data obtained in our cohort, we decided to identify online genotyping and expression datasets for STS. Unfortunately, databases are limited. The most relevant dataset to date is TCGA-SARC [50] and we decided to perform a bioinformatics analysis in order to compare data of our cohort with published data. Clinical and experimental data from 164 patients with STS were downloaded from TCGA-SARC (tissue samples with matched germline data). All types of genetic variants are reported in this dataset and out of 164 samples analyzed, only 8 patients (5%) were found to be altered on ERCC1, ERCC2 or ERCC5 genes, and of these only 1 presented an alteration in a SNP(rs34292397) of ERCC5, that we did not evaluatein our study, as reported in Table S1.

Table S1. Genetic variants in ERCC1, ERCC2 and ERCC5 registered in TCGA-SARC dataset.

Sample ID	Mutation	Gene
**TCGA-DX-A6B9**	Amplification	ERCC1; ERCC2
**TCGA-DX-A8BJ**	Amplification	ERCC1; ERCC2
**TCGA-DX-A8BL**	Amplification	ERCC1; ERCC2
**TCGA-DX-A3U7**	Deep deletion	ERCC1; ERCC2
**TCGA-DX-A23V**	Amplification	ERCC5
**TCGA-DX-A6Z4**	Amplification	ERCC5
**TCGA-DX-A23R**	Deep deletion	ERCC5
**TCGA-DX-AB37**	Missense Mutation (unknown significance)	ERCC5 (rs34291397)

We subsequently performed a chi squared test between histotype proportions in our cohort and TCGA-SARC samples and found that there are no significant differences (χ^2=3.94 ; p=0.27) (Figure S1).

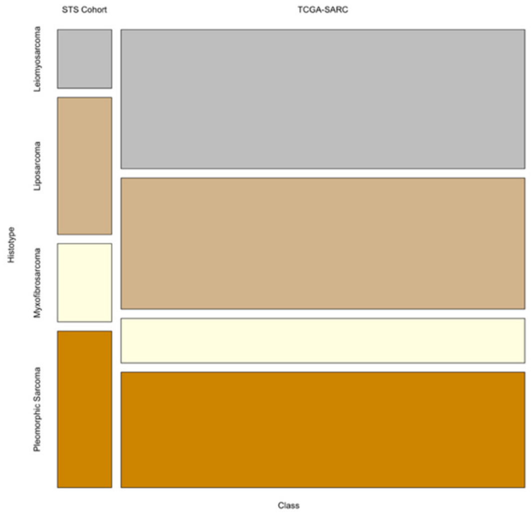

Figure S1. Comparison of histotype distributions between our patient cohort and TGCA-SARC cohort.

Thereafter we performed a chi squared test between ERCC genes expression in our cohort and TCGA-SARC samples and found that there are no significant differences in ERCC2 (χ^2=0.00002 ; p=0.99) and ERCC5 (χ^2=2.21 ; p=0.14) expression while there are significant differences in ERCC1 expression (χ^2=4.53 ; p=0.03) (Figure S2). High and Low expression levels are computed based on the

median of the expression in each group. Furthermore there are no significant differences in the overall expression of the three genes between our cohort and TCGA-SARC database (χ^2=0.42 ; p=0.52). Overall expression is considered "High" at least two genes show high expression.

A

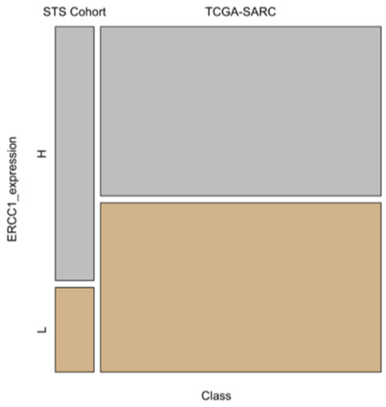

B

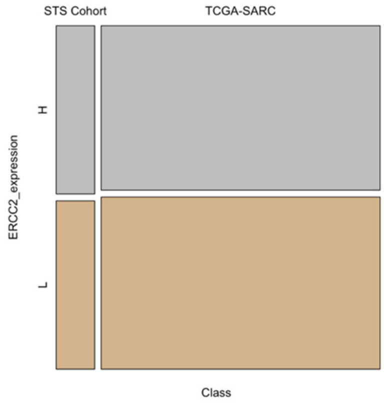

C

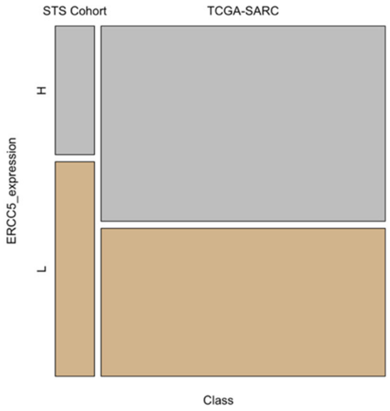

D

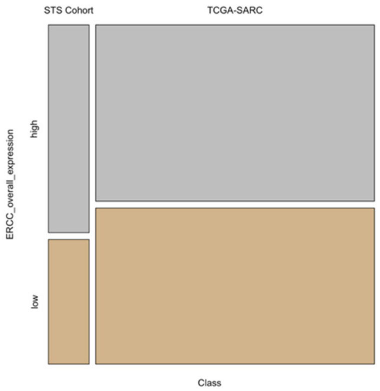

Figure S2. Comparison of ERCC genes expression between our cohort and TGCA-SARC cohort, A: ERCC1; **B**: ERCC2; **C**: ERCC5; **D:** ERCC overall expression.
Note. H, High; L, Low.

Finally, we decided to compare, as done in our cohort, expression data of ERCC genes in TCGA-SARC to clinical features: sex, grade and histotype. We constructed a heatmap to visualize this analysis, which did not provide any significant result (Figure S3).

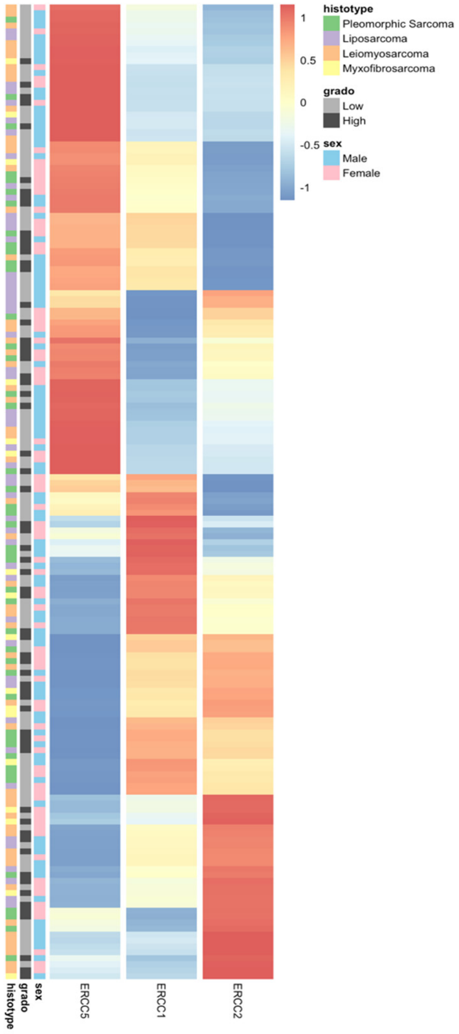

Figure S3. Expression clusters of ERCC1, ERCC2 and ERCC5 for each patient. The rows represent each sample, Sex, Histotype and Grade. The columns represent the expression of ERCC1, ERCC2 and ERCC5.
